# Complex Cerebral Artery Anomaly Rete-like Formation of the Terminal Carotid and Middle Cerebral Arteries with Bilateral A1 Segments Fenestrations

**DOI:** 10.3390/diagnostics15111333

**Published:** 2025-05-26

**Authors:** Dragoslav Nestorovic, Igor Nikolic, Andrija Savic, Drazen Radanovic, Marko Miletic, Vladimir Cvetic

**Affiliations:** 1 Center for Radiology, University Clinical Centre of Serbia, Pasterova 2, 11000 Belgrade, Serbia; 2 Clinic for Neurosurgery, University Clinical Centre of Serbia, Koste Todorovica 4, 11000 Belgrade, Serbia; 3 Medical Faculty, University of Belgrade, Dr Subotica Starijeg 8, 11000 Belgrade, Serbia; 4 Institute for Medical Pharmacology, Dr Subotica Starijeg 1, 11000 Belgrade, Serbia

**Keywords:** cerebral circulation, anomaly, rete-like, carotid artery, middle cerebral artery

## Abstract

We present a rare case of a 16-year-old male who was admitted with bilateral tinnitus and subsequently underwent magnetic resonance imaging (MRI) and digital subtraction angiography (DSA) for further evaluation. The left internal carotid (ICA) artery had a normal caliber but ended as a stump at the C7 segment, with a network of filiform vessels from both the stump and right posterior communicating artery (PComm). The right PComm was hypertrophic and the right posterior cerebral artery (PCA) was mainly supplied by the right ICA. The right ICA’s bifurcation and the initial middle cerebral artery (MCA) segment were absent, while the MCA trunk was hypoplastic. The right PCA and pial branches vascularized the temporal lobe, with collaterals between the PCA and MCA. The left ICA was slightly enlarged with double fenestration at the left A1 segment. The right A1 segment of the anterior cerebral artery had double fenestration and while several diagnoses were considered, no single diagnosis fully explained all clinical findings. A thorough review of the existing literature yielded no comparable cases, highlighting the uniqueness of this presentation. This case emphasizes the complexity of cerebral vascular anomalies and the challenges associated with diagnosing such rare conditions, underscoring the need for careful assessment.

**Figure 1:**
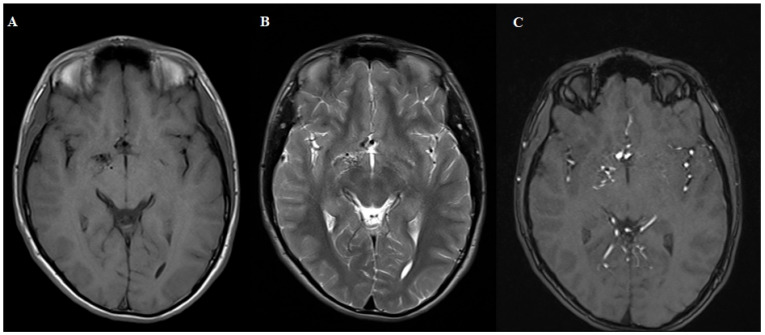
MRI T1 (**A**), T2 (**B**) and 3D TOF (**C**). Sequences demonstrating normal brain parenchyma with a tangle of small blood vessels without a visible draining vein. The patient was referred for digital subtraction angiography.

**Figure 2:**
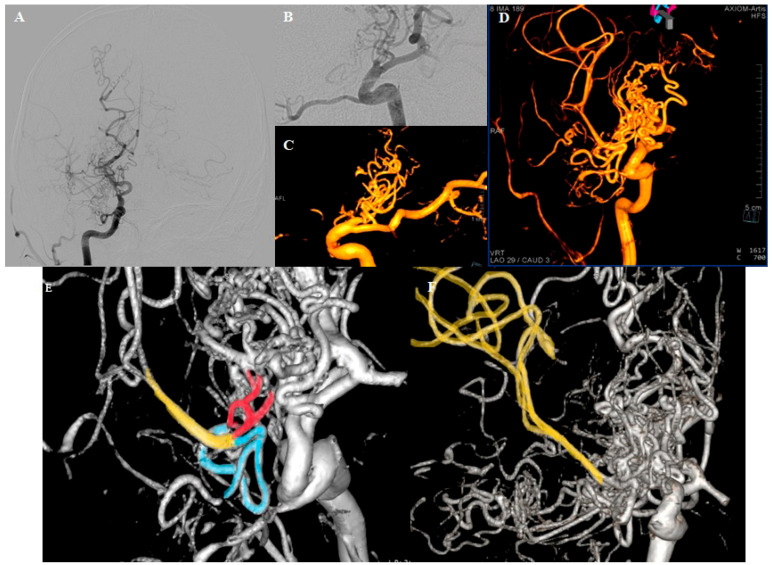
DSA. (**A**) Right ICA traced up to the end of C6 segment, where the hypertrophic PCA originates. Multiple small blood vessels originating in the projection of the carotid artery termination, forming a complex network. Right MCA is faintly shown. (**B**,**C**) Small capillary vessels rising from the “stump” of the right carotid and the PComm. (**D**) The network vessels forming the right MCA upper division; the perforators of the M1 segment originate from the described network. (**E**,**F**) Upper (red) and lower (blue) branches of the network merging to form a thin trunk of the upper division of the MCA (yellow). Our initial suspicion of an arteriovenous malformation was dismissed considering the clear absence of draining veins.

**Figure 3:**
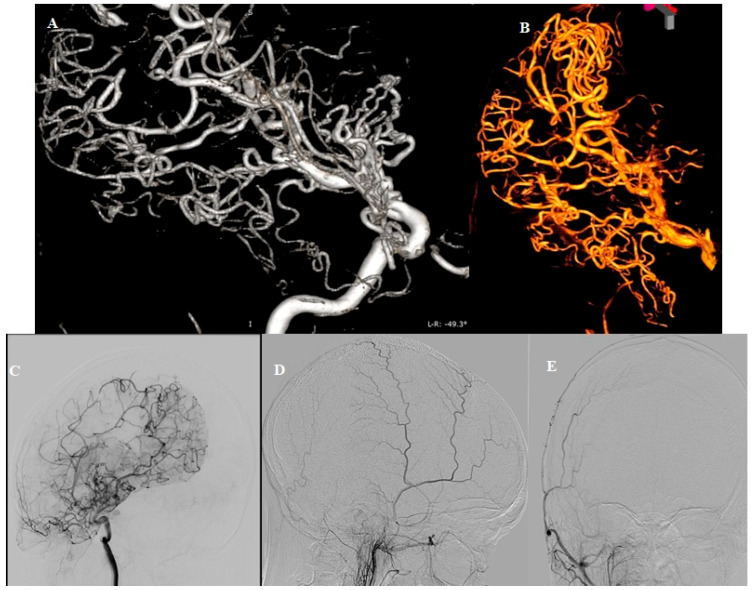
DSA (**A**,**B**) Arborization of the right PCA, taking over the vascular territory of the lower division of the right MCA. (**C**) Pial collaterals between branches of the right PCA and MCA. (**D**,**E**) Absence of the right external carotid artery (ECA) contribution. Our second consideration was Moyamoya syndrome, a condition characterized by reduced blood flow through the large vessels of the anterior circulation, prompting the development of collateral circulation near the carotid apex, cortical surface, leptomeninges and along of the branches of the ECA. Bilateral findings are also commonly observed [1]. While there are some similarities with Moyamoya disease, the angiographic morphology features observed in this case are not convincing. In Moyamoya disease, a typical “tapered” appearance of the occluded terminal portion of the carotid artery is common, with numerous collateral vessels arising nearby, demonstrating a complex angiographic course. The perforator arteries are usually hypertrophic and difficult to distinguish. In our case, the ICA terminus is blunt, with collaterals originating directly from its apex, lacking the characteristic “puff of smoke” angiographic appearance seen in Moyamoya disease. The normal presentation of the perforating arteries and the absence of dural collaterals from both the ophthalmic arteries and branches of ECAs (especially middle meningeal artery) are noteworthy. Therefore, after careful consideration, the hypothesis of Moyamoya disease was also ruled out.

**Figure 4:**
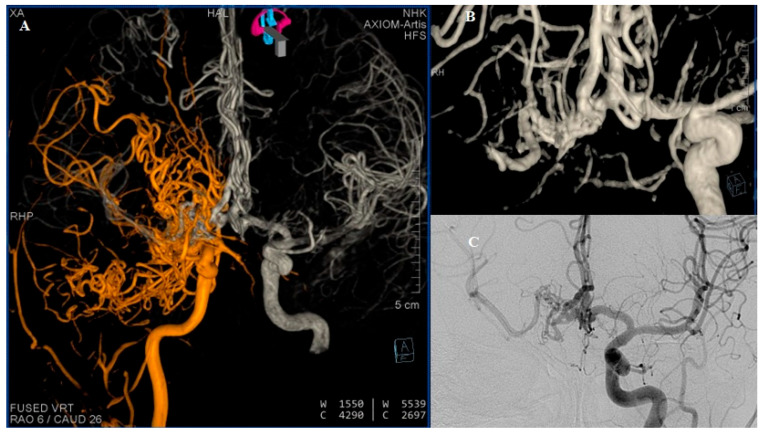
DSA (**A**) Reviewed representation of carotid circulations shown bilaterally after fusion of 3D reconstructions. (**B**) Three-dimensional reconstruction of the ACA complex showing double fenestration of the distal part of the left A1 segment and double fenestration of entire right A1 segment, where the capillary network is directly visualized. AComm and both A2 segments appear without anomaly. (**C**) The right MCA, better visualized through the contralateral carotid circulation. Many studies have reported a rare developmental anomaly which some authors refer to as “rete mirabile” or “rete-like collaterals” [2]. In 1968, Lie classified six types of collateral circulation resulting from developmental disturbances of the ICA. Type F in particular represents a mesh-like formation in the pre-cavernous segment, consisting of several small arteries that supply the cavernous segment [3,4]. The classic diagnostic criteria for rete formation include hypoplastic ICA, the presence of an arterial plexus between the internal maxillary artery and cavernous segment of the ICA, a dilated ophthalmic artery, a normal-sized supraclinoid segment of the ICA, supplied from both the arterial plexus and ophthalmic artery, bilateral lesions, and the absence of abnormal intradural blood vessels [5].

**Figure 5:**
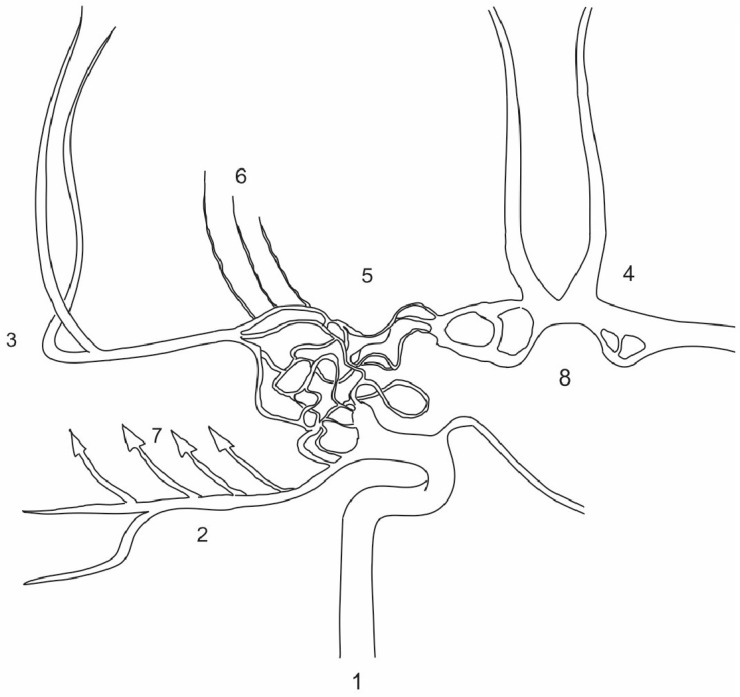
Schematic representation of the patient’s carotid basins shown bilaterally, showing the similarities to Twig-Like MCA (T-MCA) although over a broader vascular territory. T-MCA or rete-MCA represent an anatomical anomaly of unclear etiology. This condition is characterized by the absence of either a smaller or larger portion of the M1 MCA segment accompanied by non-fused primordial blood vessels that form a network connecting the proximal and distal segments of the normal MCA, while the perforator arteries originate from this network [6]. The network of blood vessels extending from the ICA to the hypoplastic M1 segment resembles what is observed in T-MCA, though it is more extensive, involving the terminal segment of ICA and part of the contralateral A1 segment. Additionally, there are multiple fenestrations on both A1 segments which are known to be a result of partial failure of fusion of paired primitive embriologic vessels or a consequence of incomplete obliteration of the primitive vascular network [7]. T-MCA is also recognized for its elevated risk of stroke, aneurysm formation and intracranial hemorrhage, as well as complications during mechanical thrombectomy [8]. Considering the similarities, these risks may be particularly applicable to the patient’s condition. 1. Right ICA; 2. right PCA; 3. right MCA; 4. ACA complex; 5. network of anomalous vessels; 6. M1 perforating branches arising from the network; 7. factors contributing to changes in the right temporal lobe; 8. bilateral fenestrations of the A1.

## Data Availability

The original contributions presented in this study are included in the article. Further inquiries can be directed to the corresponding author.
